# Challenging lymphoid malignancy of primary central nervous system lymphoma: A case report

**DOI:** 10.1016/j.amsu.2020.08.011

**Published:** 2020-08-12

**Authors:** Ganesh Kasinathan, Ahlam Naila Kori, Nurhidayah Hassan

**Affiliations:** a Haematology Unit, Tengku Ampuan Afzan Hospital, Kuantan, Pahang, Malaysia; b Histopathology Unit, Department of Pathology, Tengku Ampuan Afzan Hospital, Kuantan, Pahang, Malaysia

**Keywords:** Primary central nervous system lymphoma, Caudate nucleus, De Angelis protocol, Whole brain radiotherapy

## Abstract

**Introduction:**

Primary central nervous lymphoma is an aggressive disease without evidence of systemic spread with an annual incidence of 7 cases per 1,000,000 people in the United States.

**Case presentation:**

A 68-year-old gentleman of Malay ethnicity presented with left sided weakness associated with reduced sensation for one month. The patient was healthy and denied any constitutional symptoms, joint pains, rash or seizures. There was no recent trauma. Physical examination revealed left upper and lower limb motor grade power of 3/5 with upper motor neurone weakness of the left facial nerve. He had brisk reflexes and an upgoing extensor plantar response. Brain imaging (Magnetic Resonance Imaging) showed two lesions: one occupying the right head of the caudate nucleus and the other seen at the right side of the body of the corpus callosum. Histomorphology and immunohistochemistry confirmed Diffuse Large B-Cell Lymphoma (DLBCL) of non-germinal center type. He was treated with De Angelis protocol which involves chemoradiotherapy consisting of high dose methotrexate and whole brain radiotherapy (WBRT), followed by high dose cytarabine. Brain imaging post chemoradiation showed complete remission.

**Conclusion:**

Prompt detection with appropriate therapeutic protocol could significantly minimise the permanent neurological deficits in patients with this rare and challenging lymphoid malignancy.

## Highlights

• Primary central nervous system lymphoma (PCNSL) is an aggressive malignancy which often poses a diagnostic dilemma.
• Epstein-Barr Virus related PCNSL is more common in immunocompromised patients.
• Prompt detection minimises permanent neurological deficits in patients with PCNSL.

## 1 Introduction

Primary central nervous lymphoma (PCNSL) is a highly aggressive non-Hodgkin malignancy without evidence of systemic spread and has an annual incidence of 7 cases per 1,000,000 people in the United States [1]. The actual incidence is unknown in Asia.

PCNSL represents 4% of all intracranial neoplasms and 4–6% of all extranodal lymphomas [2]. The incidence of primary central nervous system lymphoma is much higher in immunocompromised populations. In recent years, a rising incidence particularly in immunocompetent adults older than 60 years of age occur with an incidence of 0.5 per 100,000 per year [2]. PCNSLs among immunocompetent and immunocompromised patients have differing aetiology. The occurrence of Epstein-Barr Virus (EBV)‐related PCNSL is more common in immunocompromised patients compared to that in immunocompetent individuals [3]. PCNSL often are chemoradiation sensitive unlike other types of brain tumours.

International Extranodal Lymphoma Study Group (IELSG) is a prognostic tool for distinguishing risk groups in immunocompetent patients with PCNSL. This prognostic tool comprises of five components: age, ECOG performance status, serum Lactate Dehydrogenase (LDH), cerebrospinal fluid protein and involvement of deep brain structures. The presence of adverse risk factors 0 to 1, 2 to 3 and 4 to 5 correlates inversely with 2-year survival rates: 80%, 48% and 15% respectively [4].

## 2 Case presentation

A 68-year-old male of Malay ethnicity presented to the haematology unit of Tengku Ampuan Afzan Hospital, Malaysia with a one-month history of left sided body weakness associated with reduced sensation and unsteady gait. His left sided body weakness was progressive in the last one week which lead him to seek treatment. He also complained of retrograde amnesia. B symptoms were absent. He is known to be hypertensive in which he is on oral amlodipine 10 mg daily. He previously worked with the national army as a soldier and currently in the last 15 years, he lives on an army pension. He is a non-smoker and a teetotaller. On further history, he has never consumed any traditional or recreational drugs. He has no significant family history and denies any high-risk behaviour.

On clinical examination, he was alert and orientated. His blood pressure was 126/82 mmHg with a pulse rate of 80 beats per minute. Neurological examination revealed a power grade of 3/5 over his left upper and lower limbs with hypertonia and brisk reflexes of his left elbow, knee and ankle. Signs of upper motor neuron lesion were positive in the left foot (Babinski's sign) and the left facial nerve. He showed an ataxic gait with no other cranial nerve palsies or cerebellar signs elicited. The results of other systemic examinations were unremarkable.

His complete blood count showed a haemoglobin of 13.8 g/dL, total white cell count of 6.2 × 10^9^/L and a platelet count of 220 × 10^9^/L. He had an elevated serum lactate dehydrogenase of 550 U/L. The cerebrospinal fluid (CSF) analysis parameters for protein and glucose were in the normal range. CSF cytomorphology and flowcytometry analysis did not reveal any cluster of abnormal cells.

**Fig. 1:**
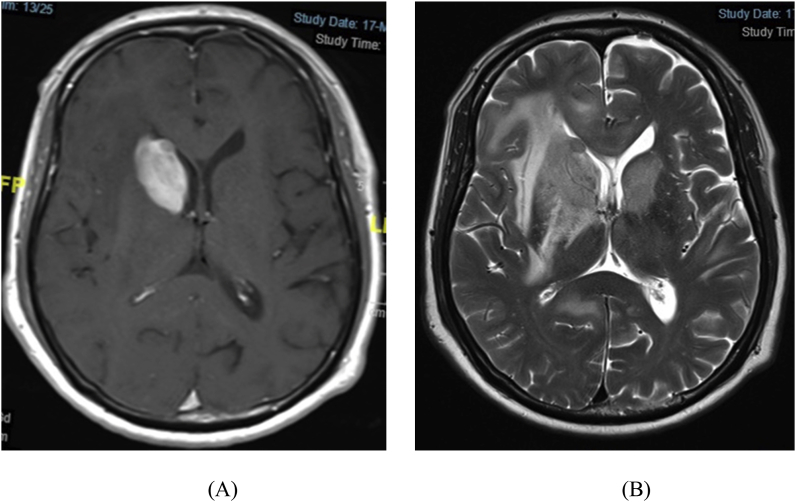
Brain MRI shows vividly enhancing intra-axial mass with irregular margins occupying the head of the right caudate nucleus. Another mass at the right side of the body of the corpus callosum extending cranially to involve the right cingulate gyrus. Significant perilesional edema with local loss of grey-white matter differentiation, no midline shift, slightly compressed right lateral ventricle with no hydrocephalus. T1-Weighted Image (A) and T2-Weighted Image (B).

The Magnetic Resonance Imaging (MRI) examination of the brain (Fig. 1) showed a vividly enhancing right intra-axial mass measuring 3.5 × 2.6 × 4.0 cm occupying the head of the right caudate nucleus. Another mass measuring 6.3 × 1.7 × 1.8 cm was seen at the right side of the body of the corpus callosum. The two masses were associated with perilesional edema. No midline shift or hydrocephalus were seen.

His baseline whole body computed tomography (WBCT) staging did not depict any enlarged lymph nodes and the bone marrow trephine biopsy did not show any infiltration. His viral hepatitis screening and HIV testing using fourth generation antigen/antibody analysis were all non-reactive. Polymerase Chain Reaction (PCR) and serology were negative for the evidence of Epstein Barr Virus infection.

**Fig. 2:**
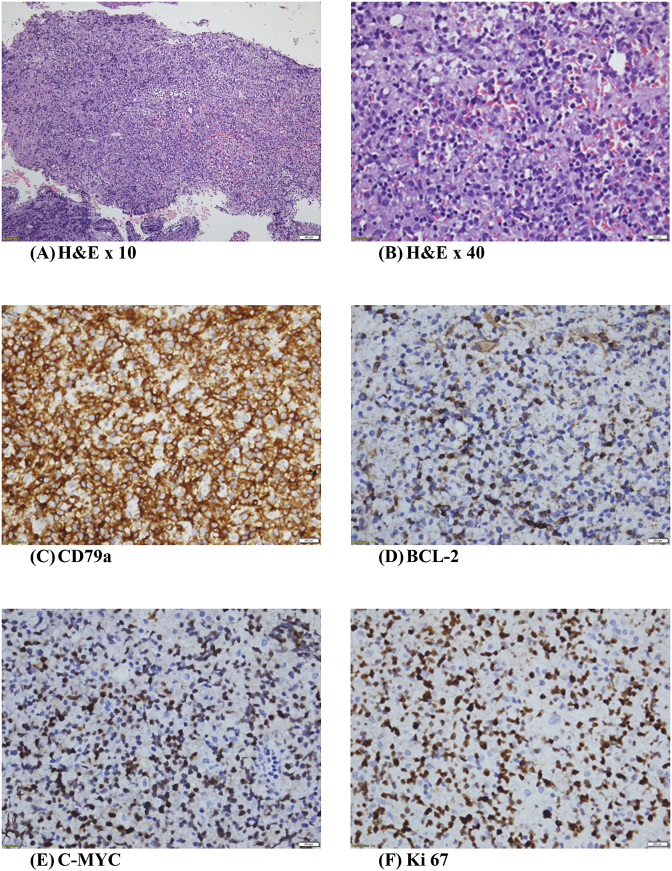
(A & B) Haematoxylin & Eosin. Brain parenchyma with infiltration by medium to large atypical lymphoid cells arranged in a diffuse pattern. The neoplastic cells are round to ovoid, having vesicular nuclei with some exhibiting prominent nucleoli. **(C**–**E)** Immunohistochemistry study with strong intense positivity for CD79a, diffuse positivity for BCL-2 and C-MYC. **(F)** Ki 67 (proliferation index) is 90%.

A stereotactic brain biopsy of the head of the right caudate and histomorphology examination (Fig. 2) demonstrated infiltration by medium to large atypical lymphoid cells arranged in a diffuse pattern. The neoplastic cells were round to ovoid, having vesicular nuclei with some exhibiting prominent nucleoli.

Immunohistochemistry (Fig. 2C–E) revealed tumour cells were positive for CD10, CD20, CD79a, BCL-2, BCL-6 and C-MYC. They were negative for Epstein Barr-virus encoded RNA 1 (EBER1). The proliferation index (Fig. 2F) was 90%.

He was diagnosed as primary central nervous system lymphoma, diffuse large B-cell lymphoma non-germinal B-center subtype with an International Extranodal Lymphoma Study Group (IELSG) prognostic score of 4 (High Risk-age >60, ECOG: 2, LDH: elevated, CSF protein: not elevated, Deep structures involved: corpus callosum).

He was treated with a 19-week De Angelis chemoradiotherapy protocol which involved high dose Methotrexate 3g/m^2^-based polychemotherapy (vincristine/procarbazine/dexamethasone) induction and whole brain radiotherapy (WBRT) consolidation of 42 Gy (21 fractions) followed by high dose cytarabine.

Following chemoradiation therapy, motor power of his left upper and lower limbs returned to normal with no residual neurological deficit. Follow-up brain MRI examination revealed a complete remission over the past 14 months. He is now on regular follow up at the haematology clinic.

## 3 Discussion

The age of this immunocompetent patient (68 years) is consistent with the reported median age at diagnosis of 60 years. However, males are less frequently affected with PCNSL with reported male:female ratio of 1.2:1.7.5 [5].

The duration of the symptoms in this patient was shorter compared to the reported focal neurological deficits, personality changes, and increased intracranial pressure which often started weeks to months prior to presentation [5].

The MRI finding of two masses in the brain of this patient represents an unusual number of lesions and locations compared to those reported which most often involved a single brain lesion either located in the hemispheres, basal ganglia, corpus callosum, and periventricular regions (66%), supratentorial location (87%), or frontoparietal lobes (39%) [6]. Less frequently, eyes (15–25%), cerebrospinal fluid (7–42%) and only in rare cases, the spinal cord is involved [6].

Other differential diagnoses include secondary brain metastases, primary gliomas, cerebral toxoplasmosis, neurosarcoidosis, and progressive multifocal leukoencephalopathy (PML).

Stereotactic-guided biopsy is essential for establishing the definitive diagnosis as well as permitting rapid detection of tumour cells intra-operatively. However, small-sized samples could have a negative impact on the diagnosis [7].

The PCNSL in this patient was in accordance with the reported 90% of PCNSLs being diffuse large B-cell lymphoma non-germinal center subtype [8]. The remaining include Burkitt lymphoma, low grade and T-cell lymphomas [8].

Treatment modalities for PCNSL have rapidly evolved. Surgical resection in PCNSL has a limited role as this disease frequently involve deep brain structures and this leads to increased risk of permanent neurological deficits. Until 1980s, the main modality of treatment was whole brain radiotherapy (WBRT). Overall response rates reached 90% but overall survival was limited to 12–18 months with WBRT alone [9]. Focal brain radiotherapy is not employed in PCNSL as this disease frequently involves deep brain structures and disseminate within CSF pathways.

PCNSL is extremely chemosensitive. The backbone of multimodal therapy for PCNSL is high dose methotrexate-based polychemotherapy. High dose methotrexate (HD-MTX; ≥ 3 g/m^2^ body surface, given as a 4-h IV infusion) is the most effective single active agent and a key component of all combination regimens [10]. Treatment response to HD-MTX/Cytosine arabinoside was further improved by adding rituximab, an anti-CD20 monoclonal antibody, to the regimen; however, the effect was only significant if thiotepa (MATRix protocol) was also added (overall response rate: 53% versus 74% versus 86%) [10]. More haematological adverse events were seen in the intensified arm, but there was no significant increase in serious infections or mortality.

The De Angelis protocol includes five cycles of high dose intravenous methotrexate (2.5g/m^2^), vincristine and oral procarbazine (MVP) with intrathecal methotrexate preceding whole brain radiotherapy (WBRT) 45 Gy and post WBRT high dose cytarabine [11]. The Overall Response Rate (ORR) reached 94% including 58% complete responses and the median progression-free survival was 25 months (with median overall survival 37 months). Neurotoxicity was reported in 15% of patients, although the duration of follow-up may not have been extensive enough to define a relatively late outcome [11]. Neurotoxicity secondary to De Angelis protocol may occur over a period of months to years which include cerebral atrophy, amnesia, leukoencephalopathy and incontinence. Despite primarily affecting patients older than 60 years, neurotoxicity was not observed in this patient over a 14-month follow-up period. Efficacy has also been shown with approaches utilizing consolidation high dose conditioning therapy (thiotepa, busulfan, carmustine) followed by autologous stem cell transplantation (HDT-ASCT) which appears to have promising data with a 2-year survival of 56% [12].

About one third of all PCNSL patients are primarily refractory to therapy and in patients with long-lasting remission after initial treatment (median 24–26 months), re-exposure to HD-MTX–containing chemotherapy proved effective with a high response rate (85–91%) and a median survival of 41–62 months [13]. If HD-MTX is contraindicated due to age or renal impairment (creatinine clearance <50ml/min), patients can be treated with a combination of rituximab and temozolomide given as 28-day cycles until disease progression, for which an overall response rate (ORR) of 47% and overall survival (OS) of 21 months was found in a retrospective analysis [13].

In refractory patients with multiple previous therapies, options, such as temsirolimus-Mammalian Target of Rapamycin (mTOR inhibitor), lenalidomide, pemetrexed and Bruton tyrosine kinase inhibitor ibrutinib, which interfere with B-cell receptor signalling and therefore have an effect on proliferation and survival of lymphoma cells, and so-called checkpoint inhibitors such as nivolumab, which modulate T cell–mediated immune response, show activity, but are associated with significant toxicity in some patients [14]. Elderly patients with relapsed PCNSL treated with ibrutinib monotherapy at 560mg/day have a median survival of 10.3 months. A combination of intravenous pemetrexed (500mg/m2), a folate antimetabolite with rituximab 375 mg/m^2^ every 21 days in the elderly (>65 years of age) appears to be well tolerated with an OS of 11.2 months [15].

Although 50% of the patients with an initial response to therapy experienced a relapse, this reported case showed response to therapy by being disease-free over a 14-month follow-up period. Many patients with recurrent PCNSL often suffer from disabling symptoms making them difficult to participate in clinical trials.

## 4 Conclusion

Primary central nervous system lymphoma remains an important clinical entity and poses a diagnostic dilemma to many clinicians. Prompt and early detection with appropriate therapeutic protocol could significantly minimise the permanent neurological deficits in patients with this rare and challenging lymphoid malignancy.

## Author contributions

G.K. analysed the data, designed the paper, and wrote the manuscript. A.N.K and N.H. made critical revisions and approved the final manuscript.

## Declaration of competing interestCOI

All authors declare no potential conflicts of interests.

## Ethical approval

Ethical approval is not required as this is not a clinical trial.

## Funding

Self-funding.

## Guarantor

Ganesh Kasinathan is the guarantor of this manuscript.

## Informed consent

Written informed consent was obtained from the patient for publication of this case report and accompanying images. A copy of the written consent is available for review by the Editor-in-Chief of this journal on request.

## Provenance and peer review

Not commissioned externally peer reviewed.
